# Towards PubMed 2.0

**DOI:** 10.7554/eLife.28801

**Published:** 2017-10-30

**Authors:** Nicolas Fiorini, David J Lipman, Zhiyong Lu

**Affiliations:** National Center for Biotechnology Information, US National Library of Medicine, National Institutes of Health, Bethesda, United States; National Center for Biotechnology Information, US National Library of Medicine, National Institutes of Health, Bethesda, United States; National Center for Biotechnology Information, US National Library of Medicine, National Institutes of Health, Bethesda, United States

**Keywords:** biomedical literature search, biomedical text mining, PubMed, scientific publishing

## Abstract

Staff from the National Center for Biotechnology Information in the US describe recent improvements to the PubMed search engine and outline plans for the future, including a new experimental site called PubMed Labs.

## Introduction

PubMed is a search engine for the biomedical literature that provides access to MEDLINE, a database that contains bibliographic information on more than 27 million articles from more than 7,000 journals, including full text for some 4 million of these articles (Sayers et al., 2011). On an average working day approximately 2.5 million users from around the world access PubMed to perform about 3 million searches and 9 million page views. Here, as PubMed celebrates its 20th anniversary, and as the biomedical literature continues to grow, we discuss how PubMed is changing to meet the needs and expectations of its users, and to take advantage of advances in web and information technologies.

Given a query, PubMed (http://www.ncbi.nlm.nih.gov/pubmed) returns the most recent articles first by default. While this reverse chronological sort helps users to find the latest articles on a particular topic or by a specific author, most PubMed users are accustomed to search engines that sort results by relevance. Indeed, over 80% of PubMed users only browse and click results on the first page, which is very similar to the behavior of most web users (Islamaj Dogan et al., 2009), even though more useful (and sometimes still recent) papers may be returned on the second page and beyond. Moreover, the need for relevance search becomes more critical in light of the growing use of small-screen devices **–** today over 20% of total PubMed usage happens on mobile platforms **–** where users tend to browse results even less.

To accommodate changing user needs, a number of new features have been added to PubMed in recent years, such as faceted search, query auto-suggest (Lu et al., 2009a) and author name disambiguation (Liu et al., 2014). In this article we describe how we intend to improve PubMed further (in terms of the search quality and also the user interface), and we present our path towards a next-generation system (PubMed 2.0), including a new experimental site called PubMed Labs.

## New relevance search algorithm

**Figure 1.**
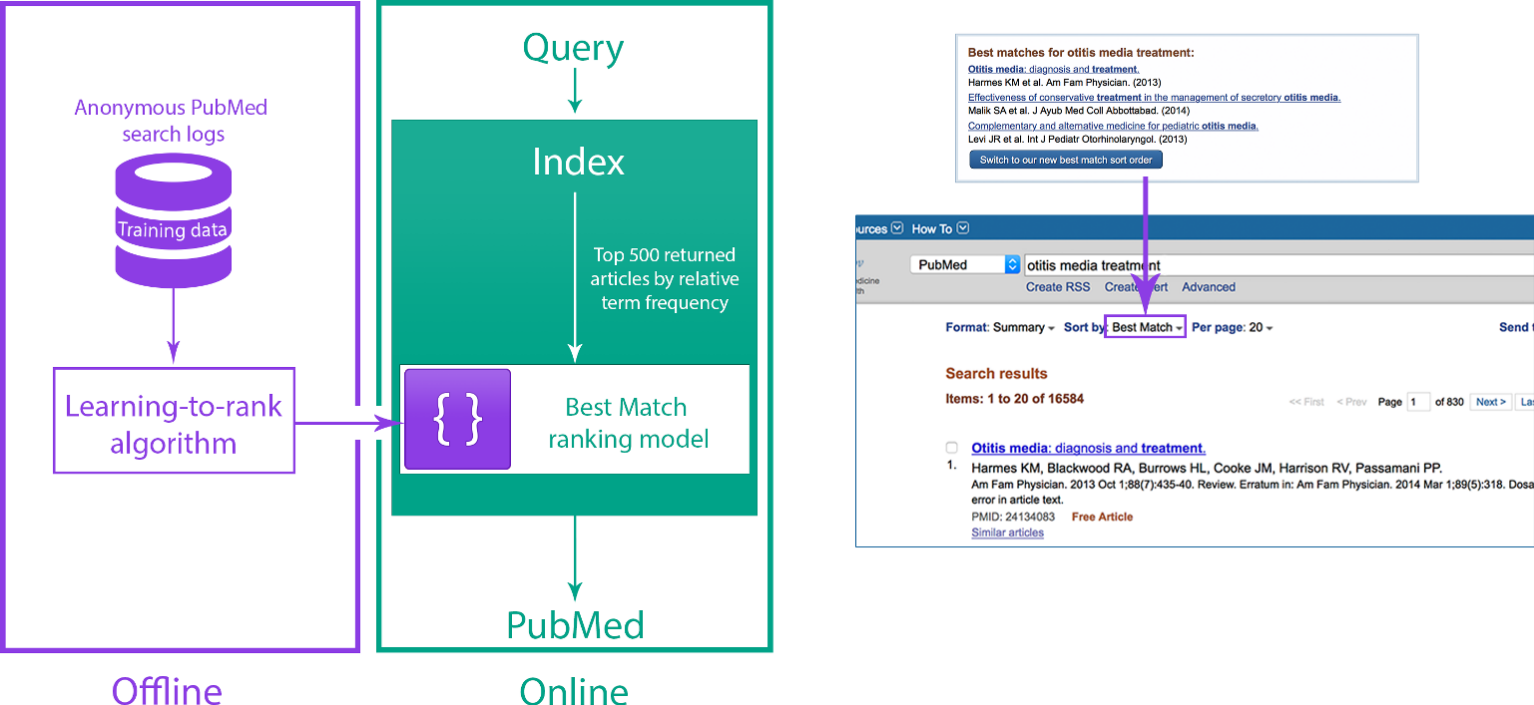
Relevance search in PubMed. (Left) System overview for the Best Match sort option in PubMed. (Right) Users are encouraged to try the Best Match sort option when PubMed detects a query for which the Best Match sort option could yield better results.

A new relevance algorithm was recently developed and deployed as one of the sort options in PubMed. It aims to provide the most relevant results and the best quality information within a fraction of a second, in response to a variety of different user information needs. This algorithm is based on a machine learning method called 'learning-to-rank' (Burges et al., 2011; Liu, 2009; Figure 1). The initial input to the learning-to-rank algorithm is the list of results returned by a classic term frequency algorithm (which ranks search results based on the relative frequency with which terms from the search query appear in the title and/or abstract of a record; Lu et al., 2009b; Robertson et al., 1996). We chose learning-to-rank because, in addition to its proven performance, it is capable of integrating a wide variety of different signals from the text record (including term frequencies) in order to return improved ranking results.

In our ranking model, we combine over 150 distinct signals to find the best results: many of these signals are computed from the number of matches between the search terms and the PubMed record, while others are either specific to a record (for example, publication type, publication date and number of abstract views within the last year) or a search (for example, the length of the query). The new ranking model was trained and tested on benchmarking data obtained from aggregated search logs.

The new ranking algorithm has been available since April 2017 to those who select the 'Best Match' sort option in PubMed, and users who select this option are 17.4% more likely to click on a link than those who select the default date-order algorithm. This figure is derived by comparing the fraction of queries with at least one article click on the first results page for the new algorithm with the same fraction for the default algorithm.

## PubMed labs and its new interface

**Figure 2.**
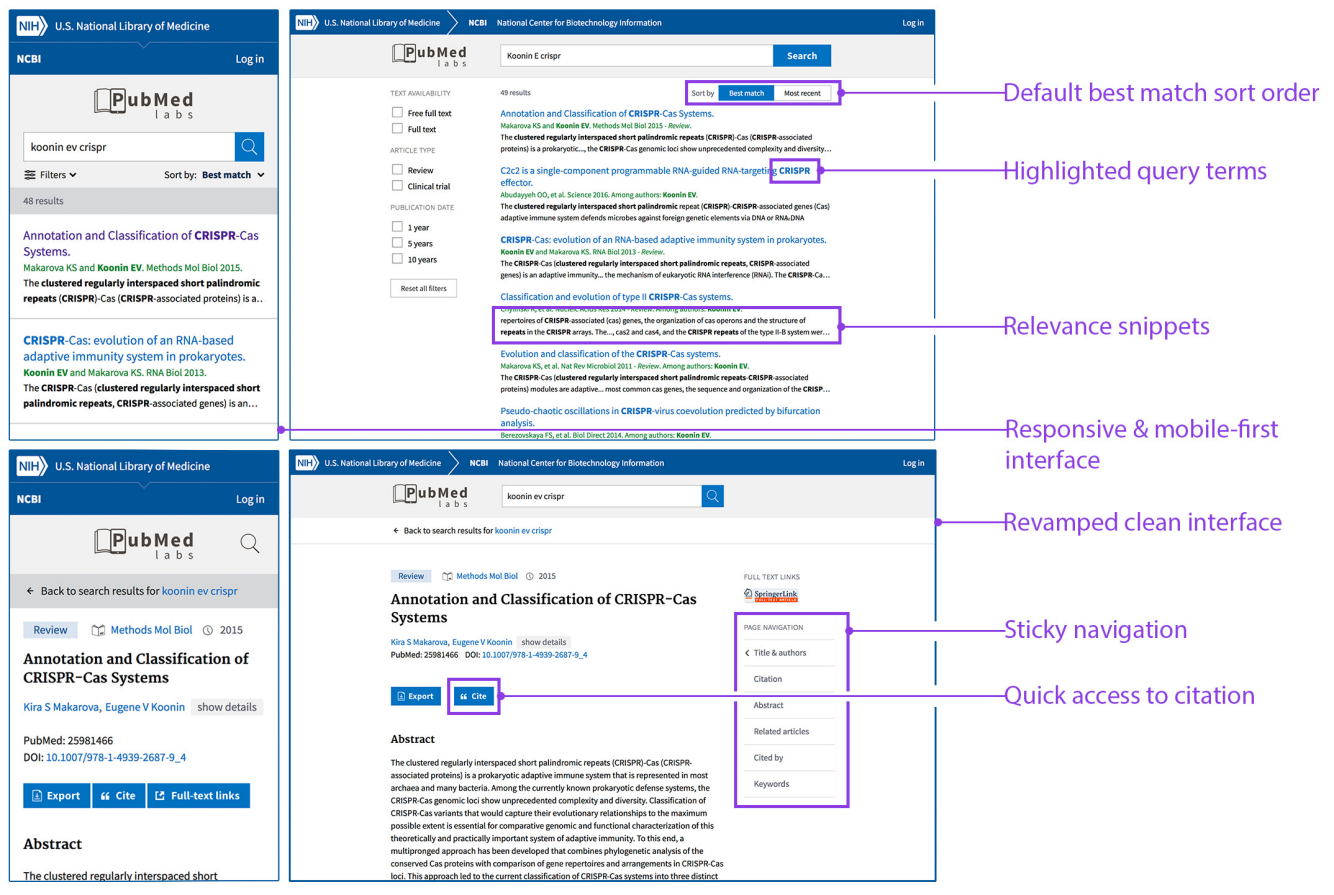
PubMed Labs: interface and features. The PubMed Labs interface on a small-screen device (left) and a desktop device (right). The default sort option in PubMed Labs is the Best Match sort option, but it is also possible to sort by date order. Each search result in PubMed Labs has search terms highlighted in the title and/or newly generated snippets, which are automatically extracted from the sentence(s) in the abstract with matching search terms. Other differences in the new interface are also highlighted.

In parallel with these developments we are launching a new experimental site called PubMed Labs (http://www.ncbi.nlm.nih.gov/labs/pubmed) that will allow us to continue our efforts to improve search quality while also working to improve PubMed's user interface: in particular, the interface for PubMed Labs (Figure 2) has been designed to provide an optimized user experience on small-screen devices (such as mobiles and tablets). We are taking a 'minimum viable product' approach in building PubMed Labs by launching it with a small number of core features and making iterative improvements and enhancements based on public testing and feedback. For example, PubMed Labs includes only a small subset of PubMed facets (selected on the basis of past usage in PubMed); decisions about the final set of facets will be based on user input and real-world usage. Other new features that we plan to introduce and analyze on PubMed Labs range from simple font color variations to complex algorithmic adjustments. Based on quantitative analysis of usage patterns, as well as direct user input and feedback, we will iteratively improve and enhance the minimum viable product.

## Outlook

As in the past, R&D will be key to the success of PubMed 2.0. However, while there has been extensive research into general web searches (e.g. Serdyukov et al., 2013), there has been less published research on usage patterns for online biomedical information resources. However, we do know that there are important differences between the two (Islamaj Dogan et al., 2009; Wilbur et al., 2006; Herskovic et al., 2007; Hersh and Voorhees, 2009). For example, PubMed users are more persistent than general web users when searching. Moreover, author names appear in over 35% of user queries in PubMed, and these names are often ambiguous because different authors share the same name and a given author can publish under multiple names (Névéol et al., 2011; Islamaj Dogan et al., 2009). This problem is made worse because authors in PubMed are traditionally represented by just their last name and first initial (e.g. Lu Z). To deal with this challenge we recently developed an automated method for author name disambiguation based on machine learning (Liu et al., 2014) and are also planning to make greater use of other related resources such as ORCIDs, regardless whether a publication has an explicit ORCID associated with it or not (most PubMed articles do not).

The rapid growth of PubMed Central (https://www.ncbi.nlm.nih.gov/pmc/) – which stores the full text of articles and is growing by more than 1000 articles per day – also presents opportunities for improving PubMed. In addition to making more content available to users, the availability of the full text (including the text in figures and tables) could lead to improved search if we can develop automated methods for coping with the increased complexity associated with full texts (Cohen et al., 2010). We are also studying the feasibility of using deep-learning technology (Mohan et al., 2017; Severyn and Moschitti, 2015; Mikolov et al., 2013) in large-scale text mining applications.

Looking into the future, we also envision PubMed serving as the central entry point for access to a range of different NCBI literature resources. To this end, we have created a new data structure, known internally as PubOne, to merge documents of different genres (such as PubMed abstracts, full texts from PubMed Central, book chapters and so on) into a single record in order to enable all-in-one access to the biomedical literature. And as we move forward from the minimum viable product, we will continue to assess and incorporate existing features such as MyNCBI into our new system. We also plan to explore if PubMed could use the outputs of various initiatives in open data and alternative metrics to improve search.

The development of PubMed has benefitted greatly from open-source platforms and technologies (such as the learning-to-rank method we used for relevance search) and from user feedback. In return for the former we would like to make our tools and techniques available to the entire scientific community via source code or web APIs. With regards to user feedback, we encourage our users and the biomedical research community to continue to use PubMed, to experiment with PubMed Labs, and to help us jointly create PubMed 2.0.
